# Prospective Relations among Low-Income African American Adolescents’ Maternal Attachment Security, Self-Worth, and Risk Behaviors

**DOI:** 10.3389/fpsyg.2017.00033

**Published:** 2017-01-24

**Authors:** Ginger Lockhart, Samantha Phillips, Anneliese Bolland, Melissa Delgado, Juliet Tietjen, John Bolland

**Affiliations:** ^1^Department of Psychology, Utah State University, LoganUT, USA; ^2^Institute for Social Science Research, University of Alabama, TuscaloosaAL, USA; ^3^School of Family and Consumer Sciences, Texas State University, San MarcosTX, USA

**Keywords:** attachment, adolescent, poverty, self-worth, substance use, violence

## Abstract

This study examined prospective mediating relations among mother-adolescent attachment security, self-worth, and risk behaviors, including substance use and violence, across ages 13–17 in a sample of 901 low-income African American adolescents. Path analyses revealed that self-worth was a significant mediator between attachment security and risk behaviors, such that earlier attachment security predicted self-worth 1 year later, which in turn, predicted substance use, weapon carrying, and fighting in the 3rd year. Implications for the role of the secure base concept within the context of urban poverty are discussed.

## Introduction

Risky behaviors such as substance use and violent acts are serious public health concerns affecting adolescents in general and impoverished minority youth in particular (Myers, 2010; Eaton et al., 2012). Though the problem has been widely studied within the context of family relationships, the current body of work is largely focused on the role of parenting behaviors such as monitoring and supportiveness in predicting adolescents’ engagement in risk behaviors. An important, though far less examined aspect of parent-adolescent relationships, attachment security, may provide additional insights into the ways in which youths’ risk behaviors emerge. A growing line of research on parent-adolescent attachment suggests that attachment processes continue to matter beyond early childhood for internal psychological challenges such as depression and anxiety (e.g., Lee and Hankin, 2009; Brumariu and Kerns, 2010), but very few studies have examined the role of adolescent attachment on externalizing problems such as substance use and violence (for recent exceptions see Sarracino et al., 2011; Scott et al., 2011). Moreover, the few studies that have examined relations between attachment security and adolescent risk behaviors have generally used cross-sectional designs (see Allen et al., 2007 for an exception) and have examined only direct effects models; however, it may be useful to examine mediating mechanisms in this link. Given the current lack of work in this area, recent calls to examine the processes through which adolescent attachment can exert its influence on risk behaviors in longitudinal studies have surfaced (e.g., Chassin and Handley, 2006). The purpose of this study was to examine a longitudinal mediation model of adolescents’ attachment security on substance use and violent behavior over the span of 3 years.

### The Secure Base in Adolescence

For many youth, the onset of adolescence marks a dramatic increase in time spent away from one’s primary caregiver and more time with friends and other non-related affiliations (Collins and Laursen, 2004). School transitions, such as the transitions to junior high and high school, are marked by substantial increases in health risk behavior and other psychological challenges (Robinson et al., 1995; Office of Juvenile Justice and Delinquency Prevention [OJJDP], 2000). From a developmental perspective, this time represents a vulnerable period, in which youth are challenged to meet changing demands of new and more complex social contexts; parents must also adapt to their new roles (Bronfenbrenner, 2005). Though attachment security in early childhood has shown lasting effects on functioning in adolescence and beyond, this vulnerable period may provoke discontinuity in parent-child attachment, such that nurturing relationships in early childhood become less so in adolescence (Doyle et al., 2009; Beijersbergen et al., 2012). Consequently, this discontinuity reveals the need to examine how the changing quality of attachment relationships impacts functioning beyond infancy and early childhood. Additionally, adolescents’ increased time spent away from parents, along with the elevated complexity of their new social contexts (Steinberg, 2008), speaks to the relevance of the secure base concept for adolescents. Seen in this light, youth who have at least one parent who is attuned and responsive to their changing needs may be better equipped to manage their new roles. Indeed, adolescents who can draw on the supportiveness and sensitivity of trusted adults show greater success in making major school transitions, including reduced behavior problems and higher levels of social competence after completing the transition (Roosa et al., 2011). Little is known, however, about the specific role of youths’ attachment security on risk behaviors across the span of adolescence.

An important component of the secure base within attachment theory is the idea that the quality of this base for exploration generates children’s mental representations of themselves (Bowlby, 1973, 1980). For example, a child whose attachment figures are responsive will likely develop a sense that she is a worthy member of her social group. Conversely, attachment figures who are less responsive may cultivate a maladaptive sense of self in the child. This process has been well documented among young children and early adolescents, such that attachment security is linked to self-worth and self-concept (e.g., Brumariu and Kerns, 2010; Bretherton et al., 2013; Gullón-Rivera, 2013). Several studies (e.g., Roberts et al., 1996; Wilkinson, 2004; Lee and Hankin, 2009) have identified self-concept as an important mediator between attachment security and internalizing problems, including depression and anxiety, but the lack of longitudinal research on how this pathway may operate for risk behavior is currently unknown. Below, we consider earlier research on the relation between self-worth and risk behavior.

### Internal Working Models and Adolescent Risk Behavior

As discussed earlier, the secure base is a context in which children and adolescents can develop positive internal working models of themselves (Bowlby, 1973, 1980). These models represent cognitive processes, in which an individual assigns a framework of the self that includes the degree to which one is valued and worthwhile. The relation between self-worth and internalizing problems has been well documented (e.g., Renouf and Harter, 1990; Trzesniewski et al., 2006) and is relatively straightforward to explain on a theoretical and conceptual level; individuals whose self-worth is compromised, such that their model of the self is characterized by a lack of value, may subsequently enter depressed emotional states. Researchers have also uncovered a link between self-worth and externalizing behaviors such as substance use (Geckil and Dundar, 2011) and violence (Kim et al., 2008), but the direction of effects is less clear, particularly in the case of violence (Wolff and Ollendick, 2006; Ostrowsky, 2010). The weaker support for the relation between self-worth and risk behavior has led to discussion about whether the effect occurs in the opposite direction, such that engaging in risky behaviors reduces self-worth, possibly due to the consequences of the behaviors, such as alienation from peers and institutionalization (Kofler et al., 2011). Unfortunately, very few studies have examined these effects longitudinally. In this paper, we argue that earlier levels of self-concept predict later risk behaviors for two reasons. First, stemming from the core idea in Attachment Theory that internal working models are the result of attachment representations, which, in the case of primary caregivers and their offspring, originates with their relationship, then representations of the self should precede subsequent risk behavior. Evidence of this temporal ordering has been documented in the literature examining self-worth as a mediator in the relation between attachment security and depression (Lee and Hankin, 2009). Second, we suggest that self-worth acts as an operating system from which individuals make decisions that either promote or inhibit their well-being. From this perspective, youth with a negative sense of self-worth view their health and safety as not worthy of preserving, ultimately leading to risky behavior.

### Poverty as a Context for Understanding Adolescent Attachment

In this prospective study, we examined the extent to which self-worth mediates the relation between adolescent attachment security and risk behaviors (specifically substance use and engagement in physical fights) among impoverished African American youth ages 13–17. Attachment processes within this group are important to study for several reasons. First, the onset of adolescence for impoverished African American youth is associated with a more substantial increase in opportunities to engage in and sustain risky behaviors than their peers in other racial/ethnic groups. For instance, data from Monitoring the Future (MTF) show that low-income African American youth are significantly more likely to engage in drug and alcohol use by the age of 14 than their Anglo and Latino peers (Johnston et al., 1999). African American youth are also more likely to persist in their use and abuse of substances into adulthood than all other major ethnic groups in the USA (Gil et al., 2002). African American youth are also significantly more likely to exhibit clinical levels of behavior problems including aggressive behaviors (e.g., Tolan et al., 2003; Snyder and Sickmund, 2006). Elevated levels of aggression are particularly pronounced among African American males as they enter high school; the frequency of verbal and physical provocations increases substantially at this time (Centers for Disease Control and Prevention [CDC], 2012). Though African American females are less likely to engage in aggressive behaviors than their male counterparts, the past decade has seen a dramatic surge in physical aggression among minority high school age females (Office of Juvenile Justice and Delinquency Prevention [OJJDP], 2000; Centers for Disease Control and Prevention [CDC], 2012). Moreover, their school environments are often conducive to engaging in risky behaviors; low-income African American high school students report that illegal substances are easy to obtain (Wallace and Muroff, 2002); their campuses are also significantly more violent than schools dominated by middle SES students (Robers et al., 2013).

From an Attachment Theory perspective, the fact that impoverished African American youth are faced with significant social-environmental challenges far beyond the normative issues faced by other adolescents presents a special opportunity to more deeply examine the insights of Bowlby’s (1973, 1980) secure base theory. Specifically, for these youth, the process of exploration away from their attachment figures and toward a social context that may compromise their well-being means that the quality of the secure base is even more critical. Adolescents who are protected by the knowledge that they can retreat from a challenging social environment and into a nurturing and responsive relationship with a caregiver are more likely to demonstrate a wide range of positive social competences than their peers without this need fulfilled (Dykas and Cassidy, 2011). Because the secure base may be particularly salient for youth in high-risk environments, we expected that the effects of attachment security on younger adolescents would persist as they grew into later adolescence.

### Summary and Hypotheses

**FIGURE 1:**
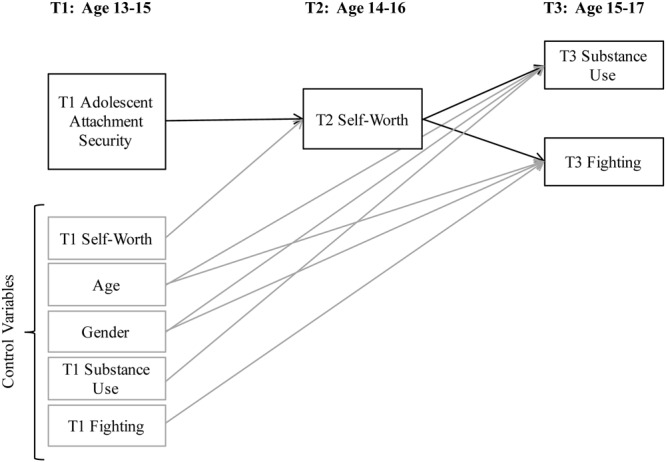
**Hypothesized model**.

The purpose of this study was to determine the prospective meditational relations among African American adolescents’ attachment security with their mothers, self-worth, and risk behaviors. These relations are important to examine because (1) relatively little is known about the pathways from attachment to risk behaviors in adolescents; (2) the prospective role of internal working models of the self in risk behaviors is currently unclear, and (3) uncovering attachment processes among low-income African American adolescents has the potential to yield useful results for prevention programs for this group. **Figure 1** shows the hypothesized model, in which attachment security at ages 13–15 predicts self-worth at ages 14–16, which in turn predicts substance use, fighting, and weapon carrying at ages 15–17. Specifically, we predicted that, based on Bowlby’s (1973, 1980) theoretical assertion that the quality of children’s secure base contributes to their mental representations of themselves, the longitudinal relation between Attachment Security and Self-Worth 1 year later would be positive, such that higher levels of Security would predict higher levels of Self-Worth. Because this representation of the self generates youths’ frameworks for making decisions that may either harm or enhance their well-being, we further hypothesized that Self-Worth would be negatively related to Substance Use, Fighting, and Weapon Carrying at Time 3. Though, we did not specify *a priori* any gender differences in the hypothesized relations, the equality of the paths across male and female participants were also tested in a separate analysis.

## Materials and Methods

### Participants

Data come from 901 African American adolescents who participated in the ‘Mobile Youth Survey’ (MYS), a longitudinal, multicohort project of predominately African American youth living in Mobile, Alabama (for details, see Bolland et al., 2013). Participants in the larger study were 12,387 predominantly African American youth (98%) age 10–18 residing in one of the 13 poorest neighborhoods in the Mobile Statistical Area. For the purposes of the current study, only data for African American participants between the ages of 13 and 15 at the first measurement occasion for which all targeted variables were measured were used to test the hypothesized model. Because the attachment security measure was added at the ninth annual wave, the first measurement occasion for the current study was year 9. Of the 1739 eligible and recruited participants age 13–15 in year 9, 1029 cases supplied parental consent, youth assent, and participated in the survey. The final sample consists of African American youth who met the age range requirement (13–15) in year 9 of the study and who had data for the independent variable, *N =* 901. Slightly less than half (47.8%) of our subsample was female, 40% lived in public housing, and the majority (66%) lived in single mother households. The median household income for our subsample was $12,157.

#### Sampling and Recruitment

In the summer of 1998 (the 1st year of data collection for MYS), approximately half of the apartment units in public housing neighborhoods with records indicating the presence of youth age 10–18 were randomly selected for recruitment. Additionally, approximately half of the non-public housing apartment units and houses were randomly selected for contact. Project recruiters attempted to make contact with primary caregivers, first by telephone, then in person, in order to determine eligibility and desire to participate. When caregivers’ written consent was obtained, interviewers scheduled a time for the youth participants to attend a group survey administration at a local church, school, or community center within walking distance of the youths’ homes. Youth who agreed to participate provided written assent. Youth who were not actively recruited by study staff, but who expressed a desire to participate, were also included if they met the criteria.

### Procedures

Once recruiters obtained informed consent of the youths’ primary caregivers, participants were invited to complete a group survey at a local church or community center. Data collection took place annually during the summer. For participants who were unable to attend a group survey, interviewers administered surveys to them individually in their homes. Youths’ assent to participate was obtained at the beginning of the survey. All surveys were read aloud by trained interviewers and participants marked their answers on a multiple-choice bubble sheet. Participants in the group setting who appeared to be having difficulty with the survey were placed in a separate room to complete the survey in either a small group or individually with an interviewer. During the years of data collection for the subsample in the current study, the participants were paid $15 at each wave.

### Measures

#### Demographics

Control variables in this study include youths’ age at time 1 (the respondents were between the ages of 13 and 15 at the first measurement occasion and then completed two additional annual assessments) and gender (male = 0; female = 1).

#### Adolescent-Mother Attachment Security

Youth responded to an adapted 14-item version of the Kerns Security Scale (Kerns et al., 1996, 2001), which assessed four aspects of attachment security: (1) Maternal Attunement (e.g., “How sure are you that your mother knows what you need from her?”; (2) Perceptions of Support (e.g., “I do/don’t like telling my mother what I think”); (3) Relationship Reaffirming Behaviors (e.g., “When I am angry, my mother tries to be understanding”), and (4) Trust (e.g., “My mother will/won’t be there for me when I need her”). Attunement items were scaled from 0 (Not at All Sure) to 2 (Very Sure); Perceptions of Support items (Do/Do Not), Relationship Reaffirming Behavior Items, (Agree/Disagree), and Trust Items (Will/Will Not), were each binary and coded 0/1. Though this scale was originally used and validated on middle childhood and young adolescent samples (Kerns et al., 2000; Kim et al., 2015), recent work has shown evidence for predictive and convergent validity in middle and older adolescents (Van Ryzin and Leve, 2012; Kerns et al., 2015). Exploratory factor analyses on these items did not distinguish these facets from one another and they are therefore aggregated into a single scale; Cronbach’s alpha for the aggregated scale was 0.70.

#### Self-Worth

The General Self Worth subscale of Harter’s (1982) Perceived Competence for Children was used to measure adolescents’ self worth. Adolescents chose one of two response choices (“like” or “don’t like”) to six statements such as “I usually [like/don’t like] the way I behave” and “I usually [like/don’t like] the kind of person I am.” Though this measure was originally constructed on a four-point scale, the current study employed binary items in order to maintain simplicity of the survey battery. Our previous work using this adapted version of the scale has demonstrated appropriate construct validity despite the binary split (Fedorowicz et al., 2014). Because the items were binary, they were scaled by creating a count of the items, with higher scores indicating higher self-worth; reliability statistics are therefore not reported.

#### Risk Behaviors

Adolescent Risk Behaviors were measured using items devised by an NICHD cooperative agreement which was funded to study adolescent health behavior among inner city youth in seven cities, including Mobile (Browne et al., 2001). The specific behaviors included in this study included substance use, fighting, and weapon carrying. Each risk behavior measure consisted of three self-reported questions that targeted both the quantity and persistence of the behavior in question. Specifically, adolescents were asked to recall whether they performed each behavior within the past year, 30 days, and week, and whether the behavior was performed either once or more than once in each time period (e.g., ‘No’; ‘Yes, just once’; ‘Yes, more than once’). The items included ‘In the past year/30 days/week, did you: (1) get drunk or high? (2) get in a physical fight? (3) carry a knife or gun?’ for substance use, fighting, and weapon carrying, respectively. For each of the risk behaviors in the model, the corresponding items (three items per behavior) were formed into an 8-point ordinal scale, such that high scores reflected greater quantity (i.e., engaging in the behavior more than once) and more recency (i.e., performing the behavior more recently). **Table 1** shows the categories of each behavior.

**Table 1:** Descriptive statistics of study variables.

	Mean (*SD*)	Minimum	Maximum
**Continuous variables**			
T1 attachment security^∗^	1.07 (0.24)	0	1.28
T1 self-worth	3.23 (1.02)	0	4.00
T2 self-worth	3.27 (1.04)	0	4.00
**Categorical variables**			
T1 age
13	288 (32.0%)
14	288 (32.0%)
15	325 (36.1%)
Female	431 (47.8%)

**Ordered categorical variables**	**T1 percent**	**T3 percent**

Substance use			
Never	75.40	67.3	
Not in past year^+^	3.60	4.5	
Once in past year	4.00	3.9	
>Once in past year	2.00	2.0	
Once in past month	2.10	3.7	
>Once in past month	1.90	1.0	
Once in past week	5.00	6.1	
>Once in past week	5.90	11.6	
Fighting			
Never	12.50	18.90	
Not in past 90 days^+^	39.90	40.60	
Once in past 90 days	12.20	12.40	
>Once in past 90 days	4.00	1.400	
Once in past month	20.60	15.90	
>Once in past month	10.80	10.80	
Weapon carrying			
Never	54.60	45.50	
Not in past 90 days^+^	12.80	18.80	
Once in past 90 days	3.70	4.10	
>Once in past 90 days	0.80	1.40	
Once in past month	6.40	4.90	
>Once in past month	1.00	1.80	
Once in past week	10.20	11.80	
>Once in past week	10.50	11.80	

*^∗^The aggregated Attachment Security measure is comprised of a combination of binary and continuous items; this is reflected in the maximum score of 1.28. ^+^The second category of each of the ordered categorical variables indicate the proportion of youth who had engaged in a given behavior at some point during their lives but not in the past year or 90 days as shown in the third category.*

## Results

### Analytic Strategy

The structure of our hypothesized model was specified according to a theoretically based approach to testing mediation models (Lockhart et al., 2011), such that the independent variable (Time 1 Attachment Security) predicts a baseline-controlled mediator (Time 2 Self-Worth), which, in turn, predicts a baseline-controlled outcome (Time 3 Substance Use and Fighting). This model specification is useful because it increases the chance of establishing temporal precedence. We used ordinal logistic path analysis to simultaneously specify the three mediation processes under examination. The specific indirect effects of youths’ attachment security on the three risk categories (substance use, fighting, and weapon carrying) were evaluated using confidence intervals generated by the bias-corrected bootstrap method described by MacKinnon et al. (2004) using 5000 draws. This computer intensive method of testing mediation has demonstrated less bias than methods using single samples because it generates confidence limits which reflect the asymmetric distribution of the mediated effect (MacKinnon et al., 2004; MacKinnon, 2008). Analyses of the hypothesized model were performed with the Mplus software package (Muthén and Muthén, 1998-2010). Missing data were handled using full information maximum likelihood under the missingness at random assumption (MAR; Enders, 2010). Documented evidence for MAR in the MYS data include (1) no associations between risk behavior variables and attrition based on regression analyses in which each outcome is regressed on a binary missingness variable and (2) sample representativeness of the larger community as measured by standardized test scores and school disciplinary records (Bolland, 2012).

### Missing Data, Descriptive Statistics, and Correlations

As can be expected in a low-income sample with a substantial number of participants living in impoverished conditions, attrition from years 1 to 3 of the current analysis was somewhat high, at 38%. Attrition was due primarily to relocation; only 7.7% of losses to follow-up were due to refusals to participate. The descriptive statistics of each of the study variables are presented in **Table 1**. In general, youth reported moderately high levels of attachment security and self worth; a preliminary MANOVA revealed that there were no gender differences for these two variables. The youth showed increasing frequency/persistence levels of both substance use and weapon carrying but slightly decreasing levels of fighting between years 1 and 3. Each of the significant correlation results (see **Table 2**) of the substantive paths of interest were consistent with the directions specified in our hypothesized model, such that attachment security at Time 1 was positively associated with self-worth at Time 2, which was negatively correlated with levels of substance use, fighting, and weapon carrying at Time 3. We further examined these relations with the path model results described below.

**Table 2:** Correlations among study variables.

	1	2	3	4	5	6	7	8	9	10	11
(1) T1attachment	-	0.112ˆ*	0.120ˆ*	0.025	-0.037	0.025	-0.038	0.025	-0.037	-0.051	-0.031
(2) T1 self-worth		–	0.484ˆ*	-0.201ˆ*	-0.188ˆ*	-0.224ˆ*	-0.174ˆ*	-0.248ˆ*	-0.189ˆ*	0.001	0.052
(3) T2 self-worth			–	-0.105ˆ*	-0.197ˆ*	-0.118ˆ*	-0.177ˆ*	-0.141ˆ*	-0.241ˆ*	0.032	0.002
(4) T1 substance use				-	0.262ˆ*	0.224ˆ*	0.142ˆ*	0.346ˆ*	0.159ˆ*	0.154ˆ*	-0.120ˆ*
(5) T3 substance use					–	0.140ˆ*	0.340ˆ*	0.265ˆ*	0.422ˆ*	0.001	-0.116ˆ*
(6) T1 fighting						–	0.204ˆ*	0.338ˆ*	0.133ˆ*	-0.058	-0.151
(7) T3 fighting							–	0.267ˆ*	0.416ˆ*	-0.109ˆ*	-0.162ˆ*
(8) T1 weapon								–	0.309ˆ*	0.068ˆ*	-0.148ˆ*
(9) T3 weapon									–	-0.038	-0.075
(10) T1 age in years										–	-0.001
(11) Gender (female = 1)											–

*T1 = Time 1; T2 = Time 2; T3 = Time 3. Age at T1 was entered as a control variable. ^∗^*p* < 0.05.*

### Path Model Results

**FIGURE 2:**
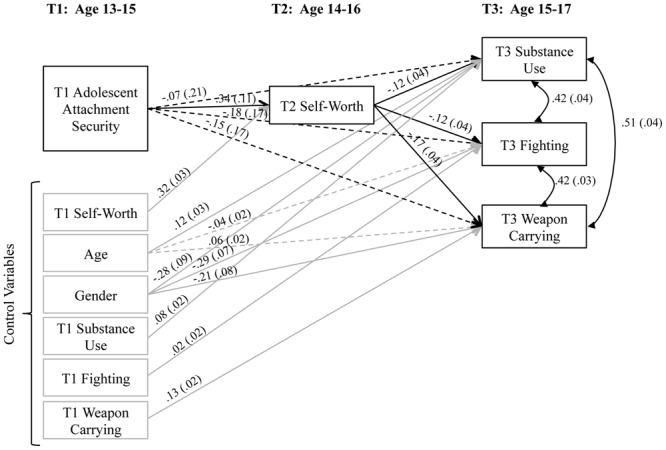
**Unstandardized model results with beta weights and (standard errors)**. Dotted paths indicated non-significant results.

Because few established measures of global model fit are available for ordinal logistic regression path models, we report the RMSEA, CFI, and the weighted root mean square residual (WRMR), which provides the mean of the covariance residuals. Our analysis showed a good fit of the model to the data according to the guidelines of Hu and Bentler (1999), RMSEA = 0.048 (<0.05 indicates a good fit), CFI = 0.959 (>0.95 indicates a good fit), and Yu’s (2002) criteria for WRMR cutoffs (<0.90 indicates a good fit because the index specifies average residuals): WRMR = 0.887. Unstandardized results, including beta weights and standard errors, of the specific paths within the model are shown in **Figure 2**. Each of the individual paths of substantive interest were significant and in the hypothesized direction.

All hypothesized mediated pathways were significant according to both normal theory and asymmetric distribution tests. The specific indirect effect from Attachment Security to Substance Use though Self-Worth was significant, with a product estimate of -0.04 (*p* < 0.05; 95% CI = [-0.10, -0.01]); the indirect effect from Attachment Security to Fighting via Self-Worth was significant, with a product estimate of -0.04 (*p* < 0.05; 95% CI = [-0.10, -0.01]); the indirect effect from Attachment Security to Weapon Carrying via Self-Worth was significant, with a product estimate of -0.06 (*p* < 0.02); 95% CI = [-0.12, -0.02]. Each of the direct paths, from Attachment Security to Substance Use, Fighting, and Self-Worth, were non-significant; adjusted direct effects = -0.07, -0.18, and -0.15, respectively, *p >* 0.05. The indirect effect from Attachment Security to Substance Use via Self-Worth accounted for 37% of the total effect (or the sum of the direct and indirect paths); the indirect effect from Attachment Security to Fighting via Self-Worth accounted for about 19% of the total effect; the indirect effect from Attachment Security to Weapon Carrying via Self-Worth accounted for about 29% of the total effect.

The test for gender differences in the fit of the model revealed χ^2^ contributions that were very close (χ^2^[26] = 27.17 and 26.82 for males and females, respectively), suggesting that the fit of the model was not different across genders; differences in individual paths were also non-significant.

## Discussion

The purpose of this study was to examine the extent to which African American adolescents’ self-worth mediated the relations between attachment security with their mothers and risky behaviors. Consistent with our hypotheses, higher levels of attachment security at age 13–15 predicted higher levels of self-worth 1 year later, which, in turn, predicted lower levels of substance use, fighting, and weapon carrying in the 3rd year. These findings extend earlier work examining similar mediating mechanisms on internalizing outcomes such as depression and anxiety (Lee and Hankin, 2009; Brumariu and Kerns, 2010) and suggest that the developmental pathway from earlier attachment characteristics to subsequent risk behavior may operate in a similar way for substance use and violence. Below, we consider the theoretical and practical implications of the results of each component of the model, followed by a discussion of the study’s limitations and implications for prevention programs.

Mother-adolescent attachment security was a significant predictor of adolescents’ self-worth 1 year later, suggesting that the quality of the secure base is important for developing and sustaining self-worth over time. Because attachment security was measured at an age (13–15) in which many youth are experiencing major social changes (e.g., Steinberg, 2008), secure attachment relationships may be a risk-reducing mechanism for this developmental stage, providing additional evidence that the secure base concept is both relevant and important for impoverished African American youth. Importantly, the youth in our sample face particularly challenging school environments, which are often plagued by inadequate resources, lack of support from teachers and administrators, and campus violence. The finding that the prospective relation between attachment security and youths’ self-worth is significant suggests that mother-adolescent attachment in early/middle adolescence may act as a risk reducer for youth who are navigating environments that could compromise their well-being. It would be interesting and important in future work to closely examine, for example, the extent to which school transitions provoke change vs. stability in the quality of attachment relationships, and whether any changes may then contribute to changes in self-worth after the transition.

Consistent with our hypotheses, we also found that mother-adolescent attachment security at Time 1 predicted Time 3 substance use, fighting, and weapon carrying through Time 2 self-worth. It is interesting to note that the strength of the mediation process with fighting as an outcome, though significant, was relatively modest in relation to the other two risk behavior variables. This result could reflect the fact that physical fights in urban, impoverished communities are nearly commonplace in adolescence and naturally reduce with time (as seen in our sample). Because weapon carrying is a more rare and serious offense, this aspect of violence could be more strongly affected by youths’ self-worth.

Our findings with regard to substance use and weapon carrying could be explained by the idea that internal working models of the self can either promote or inhibit adolescents’ health-compromising behaviors. Specifically, youths’ internal models of the self could provide a framework for making decisions that reflect the extent to which their well-being is worthy of preserving. Research on the precursors of health-related decision making shows that late adolescents who engage in risky behaviors are aware of the potential consequences of substance use and violence (for a review, see Steinberg, 2008), which runs counter to the common idea that youth of this age are not capable of evaluating their choices. We suggest, therefore, that youth with compromised self-worth are also aware of the consequences of their risky behaviors, but they have a working model of the self that promotes self-defeating behavior. Below, we consider the specific pathways for substance use and violence.

The longitudinal effect of attachment security on substance use was mediated through self-worth, such that the direct effect was non-significant. Adolescence is a time in which youths’ cognitions become more complex, such that they begin to respond to their thought processes by developing strategies to manage them. Youth with poor self-concepts may use maladaptive coping strategies to escape from the painful emotions that accompany their compromised internal working models. Indeed, substance use is a common avoidance mechanism in response to a variety of psychological challenges (Cooper et al., 1995), including *threatened egotism*, or one’s perceptions of self-worth being threatened by others in a given context (Luhtanen and Crocker, 2005). Though threatened egotism reflects a contextual phenomenon and is distinct from trait-level self-worth, it may be important to further explore it within the context of attachment relationships. Youth with low trait self-worth, for example, may also exhibit intense state fluctuations in self-worth across various contexts and domains of functioning, such that they have high feelings of self-worth as a member of their peer group, but low self-worth as a student; these levels may also fluctuate over time. This reasoning is consistent with Crocker and Wolfe’s (2001) *contingencies of self worth*, which acknowledges both global and situational sources of worth, and proposes that individuals stake their situation-specific evaluations based on their perceived importance of a given domain (e.g., peer relationships or physical attractiveness). When one invests self-worth very intensely in a domain that requires the approval of others, they are more vulnerable to threatened egotism (Crocker and Wolfe, 2001), a process that has been implicated in alcohol use (Luhtanen and Crocker, 2005).

Consistent with our hypothesis, we also found a fully mediated effect of the longitudinal associations among attachment security, self-worth, and both fighting and weapon carrying. As discussed earlier, research on the relation between self-worth and violence has yielded somewhat inconclusive findings. Our results suggest that youths’ internal working models of the self may contribute to their constructions of models of their relationships with others, ultimately impacting their tendencies for violence toward others. Attachment research could also benefit from additional explorations on the role of contingencies of self worth and threatened egotism in the pathway from attachment to violent behavior. Adolescents with poor trait-level self-worth may experience vulnerability to threatened egotism because the deficit of their internal model of the self creates inflated self-worth contingencies. Youth in disadvantaged communities may be particularly likely to invest much of their self-worth on their peers’ approval and therefore be more vulnerable to threatened egotism within the peer context, which has been linked to violence and aggression in both adolescents and adults.

Overall, the complete model illustrates the transmission of the quality of mother-adolescent attachment security to youths’ working models of the self, and ultimately their risk behaviors, across 3 years in a sample of low-income African American youth. This longitudinal study sheds light on the developmental salience of the secure base concept for middle and late adolescence. Moreover, the current study identified self-worth as a potentially critical component of adolescents’ internal working models of the self, such that self-worth acts as an operating system through which youth make choices that affect their well-being. Attachment style has been linked to contingencies of self-worth (Park et al., 2004), but researchers have yet to examine the extent to which attachment can explain trait self-worth and situational shifts in worth across domains.

The results of our study should be considered in light of a few limitations. First, the design of the study only included self-reported responses of adolescents, potentially contributing to shared method bias. Attachment security is a particularly complex construct to measure and usually requires a validated observational component such as the Adult Attachment Interview (George et al., 1985, Unpublished) to serve as a gold standard instrument. Though, we measured multiple facets of attachment security in our study, we were unable to include the marker *deidolization of mother* because it cannot reliably be assessed with self-report. Second, though the study was longitudinal and contributes to our understanding of the developmental salience of attachment security throughout middle and late adolescence, it reflects only 3 years of annual assessments, and we therefore could not capture increments of change between measurement occasions. Third, though we examined an understudied group of adolescents, the findings cannot be generalized beyond low-income African American communities. Finally, this study was limited to the attachment experiences of mother-adolescent relationships; the role of attachment security in other important relationships (e.g., fathers and peers) in predicting the mechanisms studied here should be examined in future research.

Despite these limitations, this study joins the modest number of investigations of the longitudinal mechanisms through which attachment security transmits its effects on disadvantaged adolescents’ risk behaviors. The results of the model suggest that prevention programs designed to compensate for youths’ compromised attachment relationships would likely benefit from an adult mentorship component that would form earlier in adolescence. Mentorship programs for economically disadvantaged youth may improve both self-worth (DuBois and Silverthorn, 2005) and parent-adolescent relationships (Chan et al., 2013). However, successfully targeting youths’ internal working models is a significant challenge due to the relative stability of attachment security across adolescence (Allen et al., 2004). This challenge is not likely overcome with standard school-based mentoring programs, which are the most commonly implemented mentoring programs in the USA (Karcher and Herrera, 2007). Indeed, school-based mentorship programs have demonstrated modest or no utility in changing youths’ self-worth and self-esteem (Herrera et al., 2011; See Wood and Mayo-Wilson, 2012 for a review). This lack of robustness may be attributable to subpopulations of youth who have particularly compromised relationships with attachment figures (Schwartz et al., 2011), and who may therefore need a more intensive approach such as attachment-based therapeutic treatments (Diamond et al., 2016). For these youth, the best chance of reconstructing their internal working models would likely involve implementing programs that are both long in duration (Grossman and Rhodes, 2002) and high in dose (Wood and Mayo-Wilson, 2012).

## Ethics Statement

University of Alabama Internal Review Board Utah State University Internal Review Board Parents’ consent was obtained first by visiting with them face-to-face in their homes, explaining the details of the study, and the potential risks. Youth assent was obtained at the study site. Interviewers explained the study, indicated that the youth could discontinue at any time without penalty, and described the compensation. The participants were low-income African American youth. Additional protections for this group included having African American interviewers on the team, making clear that their privacy would be protected with regard to their responses on illegal behaviors (e.g., weapon use and drugs), and providing information about community resources at the end of their participation.

## Author Contributions

GL conceived of the research question, wrote the main parts of the introduction and discussion, and performed and reported the main analyses. SP contributed to the introduction and discussion and wrote the method section. JB is the original PI of the project and conceived of the original project idea and contributed to the results and discussion sections. AB performed descriptive analyses and documented them in the results section. MD and JT refined the writing of the final drafts and filled holes in the literature review.

## Conflict of Interest Statement

The authors declare that the research was conducted in the absence of any commercial or financial relationships that could be construed as a potential conflict of interest.
